# Macrotroponin interference and association with cardiotoxicity in patients receiving cardiotoxic breast cancer therapy: a pilot study

**DOI:** 10.1186/s40959-025-00314-9

**Published:** 2025-02-14

**Authors:** Andrea Soosaipillai, Inbar Nardi-Agmon, Davor Brinc, Anselmo Fabros, Peter A. Kavsak, Paaladinesh Thavendiranathan, Ashley Di Meo

**Affiliations:** 1 Department of Laboratory Medicine and Pathobiology, University of Toronto, Toronto, Canada; 2 Division of Clinical Biochemistry, Laboratory Medicine Program, Toronto General Hospital, University Health Network, 200 Elizabeth Street, Toronto, ON M5G 2C4 Canada; 3 Department of Medicine, Division of Cardiology, Ted Rogers Program in Cardiotoxicity Prevention, Peter Munk Cardiac Centre, Toronto General Hospital, University Health Network, Toronto, ON Canada; 4 Deparment of Pathology and Molecular Medicine, Division of Clinical Pathology, McMaster University, Hamilton, ON Canada; 5 Joint Department of Medical Imaging, Toronto General Hospital, University Health Network, Toronto, ON Canada

**Keywords:** ERBB2 + breast cancer, Trastuzumab, Herceptin, Anthracycline, Myocardial injury, Cardiac troponin, Macrotroponin

## Abstract

**Background:**

Cancer therapy-related cardiac dysfunction (CTRCD) is an important adverse effect in patients receiving potential cardiotoxic cancer therapies. Interpretation of cardiac troponin results can be affected by presence of macrotroponin, which can complicate CTRCD assessment. We aimed to assess whether macrotroponin is detectable in women with ERBB2 + breast cancer receiving sequential therapy with anthracyclines and trastuzumab.

**Methods:**

A total of 20 serum samples from 12 ERBB2 + breast cancer patients (median age: 55 years, range: 30–69 years) who exhibited a significant increase in high-sensitivity cardiac troponin I (hs-cTnI) from baseline to post-anthracycline (~ 2 months after therapy initiation) and/or 3-months into trastuzumab therapy (~ 5 months after therapy initiation) and/or who had at least one hs-cTnI value above the female-specific 99th percentile (hs-cTnI > 16 ng/L) and had available banked blood for analysis were included in this pilot study. Samples were analyzed using the Abbott STAT High-Sensitive Troponin-I and Roche Elecsys Troponin T hs STAT assays. Macrotroponin was detected by treating the sample with protein G and re-measuring hs-cTn. Macrotroponin presence was defined as a hs-cTnI or hs-cTnT recovery of < 40% or 85%, respectively.

**Results:**

Macrotroponin was not identified after anthracycline treatment but was present in four patients 3-months into trastuzumab therapy, two of which had hs-cTnI concentrations above the 99th percentile. None of these patients exhibited a significant reduction in LVEF and/or GLS despite having significant elevations in hs-cTnI.

**Conclusions:**

Clinicians should be cautious of benign hs-cTn elevations resulting from macrotroponin presence, as it can complicate CTRCD assessment.

**Supplementary Information:**

The online version contains supplementary material available at 10.1186/s40959-025-00314-9.

## Introduction

Breast cancer is the most commonly diagnosed cancer and the leading cause of cancer mortality in women worldwide (1,2). It is clinically divided into three main subtypes, each with distinct risk profiles and treatment strategies (2,3); hormone receptor positive/ human epidermal growth factor receptor type 2 (ERBB2; formerly HER2) negative breast cancer (70% of patients), ERBB2-positive breast cancer (15%-20% of patients), and triple-negative breast cancer (15% of patients) [1].

Advances in treatment have led to improved survival of patients with cancer but have also increased morbidity and mortality due to treatment side effects [2]. Anthracyclines and the human ERBB2 monoclonal antibody (trastuzumab) are the two anti-cancer drugs associated with the highest cardiotoxicity risk, at times causing LV dysfunction and symptomatic HF, collectively termed cancer therapy-related cardiac dysfunction (CTRCD) [3]. Left ventricular ejection fraction (LVEF) and global longitudinal strain (GLS) are used for the detection of CTRCD during and after cardiotoxic treatment [4]. Moreover, while current recommendations support baseline measurement of cardiac biomarkers to predict the risk of developing future CTRCD, including high-sensitivity cardiac troponin I or T (hs-cTnI or hs-cTnT) and natriuretic peptides (B-type natriuretic peptide [BNP] or N-terminal pro-BNP [NT-proBNP]) [5], it should be noted that uncertainty remains surrounding the ability of cardiac biomarkers to predict CTRCD risk [2, 6, 7].

In a recent meta-analysis, cTns were identified as a predictive marker for the development of cancer therapy-related LV dysfunction in patients receiving various regimens of cytotoxic chemotherapy and/or ERBB2 inhibitor therapy. The authors found that the likelihood for LV ejection fraction impairment was much higher in patients with elevated cTn compared to cTn-negative patients [8]. Yet, despite this, 48% of patients with elevated cTn levels did not develop LV systolic dysfunction [8, 9]. Interpretation of elevated cTn may be confounded by possible presence of macrotroponin (macro-cTn) [10]. Macro-cTn, which consists of troponin in complex with anti-troponin immunoglobulin G (IgG) or, to a lesser extent troponin in complex with IgM or IgA, is not uncommon, having been identified in 2–20% of individuals, with or without cardiac disease [11, 12]. While it does not appear to be pathologic, macro-cTn can complicate the interpretation of laboratory results [10, 13–15].

In a select cohort of women with ERBB2 + breast cancer receiving sequential therapy with anthracyclines and trastuzumab, the objectives of our pilot study were to (i) assess whether macro-cTn is detectable early after therapy initiation, specifically post-anthracycline (~ 2 months after therapy initiation) and 3-months into trastuzumab therapy (~ 5 months after therapy initiation), and to (ii) explore whether there is an association between the presence of macro-cTn and changes in left ventricular ejection fraction (LVEF) or global longitudinal strain (GLS).

## Materials and methods

### Study population

**Fig. 1:**
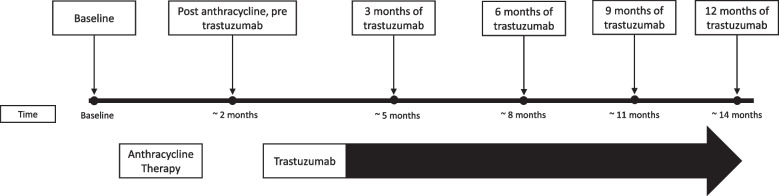
Follow-up of ERBB2 + breast cancer patients at baseline (prior to anthracycline therapy), post anthracyclines (~ 2 months after therapy initiation) and subsequently every 3 months during trastuzumab therapy (up to ~ 14 months after therapy initiation)

This study is a pilot sub-analysis of the Evaluation of the Myocardial Changes During Breast Adenocarcinoma Therapy to Detect Cardiotoxicity Earlier with Magnetic Resonance Imaging (EMBARCE-MRI) study (NCT02306538) [16, 17]. The EMBRACE-MRI study was approved by the institutional research ethics board of University Health Network (UHN) and all patients signed informed consent. Briefly, this cohort consisted of adult women with stage I to III ERBB2 + breast cancer who were scheduled to receive sequential anthracycline and trastuzumab with/without adjuvant radiotherapy and surgery. Women were recruited prospectively between 2013 and 2019 from three University of Toronto-affiliated hospitals and subsequently followed during and after cancer treatment. Specific inclusion and exclusion criteria were previously detailed [18]. Patients were followed pre-cancer therapy, post anthracyclines and subsequently every 3 months during trastuzumab therapy (up to ~ 14 months after therapy initiation) as shown in Fig. 1.

In our pilot study, amongst patients who had blood banked, we selected samples from patients that exhibited a significant increase (defined below) in hs-cTnI from baseline (prior to anthracycline therapy) to post-anthracycline (~ 2 months after therapy initiation) and/or 3-months into trastuzumab therapy (~ 5 months after therapy initiation) and/or who had at least one hs-cTnI value above the female-specific 99th percentile (hs-cTnI > 16 ng/L) as obtained from the EMBRACE-MRI study. A significant increase in hs-cTnI levels was defined as either an absolute (for baseline concentrations < 10 ng/L) or relative (for baseline concentrations ≥ 10 ng/L) difference of > 3 ng/L or > 30%, respectively [19].

Of the 136 patients recruited in the EMBARCE-MRI study, 27 patients met the inclusion criteria, from which a subset of 12 patients were included in the pilot study based on sample availability (i.e. study only started banking blood part way into recruitment, and some patients refused the optional biobanking). Specimens (serum, stored at -80 °C) available for the included patients were retrieved from the biobank for further investigation. Serum samples were available post-anthracycline and 3 months post-trastuzumab therapy initiation (*n* = 8 patients, 16 specimens), post-anthracycline only (*n* = 1 patient, 1 specimen), and 3-months into trastuzumab therapy only (*n* = 3 patients, 3 specimens). Overall, we evaluated 20 serum specimens from our cohort of 12 ERBB2 + breast cancer patients.

LVEF and GLS were used to assess cardiac function at baseline, and both follow-up time points. In each patient, 3-D full volumes (i.e., more than 20 volumes per second) of the left ventricle (LV) were obtained from the apical view and the best 3-D image was chosen for 3-D LVEF measurement using a semiautomated algorithm (4D Auto LVQ, EchoPAC Version 202 [General Electric Healthcare]). Contour adjustments were made as necessary. Studies with poor image quality were excluded. For GLS measurements, apical 4-, 3-, and 2-chamber LV images were obtained for 3 cardiac cycles (40 to 80 frames per second) and stored in an uncompressed format for analysis. Peak systolic GLS was measured using automated contours on 3 long-axis views using EchoPAC version 202 (General Electric Healthcare). Contour adjustments were made as necessary; however, after 3 attempts, poorly tracked segments were excluded. If more than 3 segments were excluded, the mean of the remaining segments was used. All analyses were performed using deidentified images by an echocardiography-trained cardiologist blinded to all clinical information, and 3-D LVEF and GLS analyses were performed independently at different time points without knowledge of the other measurements [20]. A significant reduction in LVEF was defined as a > 10% absolute reduction in LVEF from baseline, while a significant reduction in GLS was defined as a > 15% relative reduction in GLS from baseline.

### Measurement of troponin I and troponin T

Serum samples collected post-anthracycline and 3-months into trastuzumab therapy were analyzed using the STAT High Sensitive Troponin-I assay on the Alinity ci (Abbott Diagnostics, Abbott Park, IL, USA; 99th percentile, female-specific 99th = 16 ng/L) and the Elecsys Troponin T hs STAT assay on the cobas e411 (Roche Diagnostics, Laval, Quebec; 99th percentile, female-specific 99th = 9 ng/L). Baseline hs-cTnI measurements were obtained from the EMBRACE-MRI study and were performed on the ARCHITECT i2000 immunoassay analyzer (Abbott Diagnostics, Abbott Park, IL, USA).

### Macrotroponin detection

Specimens were immunoglobulin depleted using protein G GammaBind™ Plus Sepharose™ beads (GE Healthcare, Chicago IL, USA). Briefly, samples were centrifuged at 2107 × g for 10 min to remove any fibrin strands and other particulates. GammaBind Plus Sepharose in PBS (pH 7.0) was washed with binding buffer (10 mM sodium phosphate buffer (pH 7.0), 0.15 M NaCl, 10 mM EDTA) and centrifuged at 3942 × g for 3 min. This was repeated twice, for a total of three washes. A total of 400 µL of serum was added to 280 µL of washed GammaBind Plus Sepharose beads. Samples were vortexed and incubated at room temperature for 1 h on a tube revolver (Thermo Fisher Scientific, San Jose California). Samples were then centrifuged at 3942 × g for 3 min. The supernatant was collected and analyzed for troponin I, troponin T, ferritin, immunoglobulins G, A and M. Ferritin was measured using the Alinity i ferritin immunoassay and served to correct for non-specific loses after protein G treatment (i.e., ferritin was measured before and after protein G treatment) [13]. In addition, immunoglobulins G (IgG), A (IgA), and M (IgM) were measured on the Optilite (Optilite, Birmingham UK) to confirm sufficient immunoglobulin depletion following protein G treatment. Polyethylene glycol (PEG) treatment was performed to confirm the presence of macro-cTnI when possible (i.e., given sufficient serum volume). Samples were mixed with an equal volume of 25% PEG 6000 (Sigma-Aldrich) in deionized water. The mixture was incubated for 10 min at room temperature and centrifuged at 10,000 × g for 5 min. The supernatant was collected and analyzed for troponin I immunoglobulins G, A and M.

A hs-cTnI recovery of < 40% after protein G treatment was used to define the presence of macro-cTnI and < 85% for macro-cTnT as was previously described [21]. Recovery was calculated by comparing hs-cTn (hs-cTnI and hs-cTnT) concentrations before and after protein G treatment, and after volume correction with ferritin to compensate for non-specific losses [13]. Recovery (as percentage) of hs-cTn after protein G treatment was calculated as follows: (hs-cTn after protein G treatment/ hs-cTn before protein G treatment) x (ferritin before protein G treatment/ ferritin after protein G treatment). Results below the limit of detection (LoD; lowest concentration that can be distinguished from an absence of the analyte) were replaced by the concentration at the LoD for each assay (i.e., 5 ng/L for Roche hs-cTnT and 2 ng/L for Abbott hs-cTnI). A hs-cTnI recovery of < 20% after PEG treatment was used to define the presence of macro-cTnI.

To verify the 40% and 85% threshold (which has been established by only one group of investigators) [21], discarded residual plasma samples (*n* = 25) were de-identified, treated with protein G and recovery calculated. The cut-off was considered validated if 95% of recoveries were above the proposed cut-off as shown (Supplemental Fig. 1a and b). To confirm the absence of macrotroponin in the samples selected to verify the 40% threshold for macro-cTnI, a subset of de-identified residual plasma samples (*n* = 15 of 25) were analyzed using the VITROS High Sensitivity Troponin I assay on the VITROS XT 7600 (Ortho Clinical Diagnostics, Raritan, NJ, USA), which has lower immunoreactivity for macro-cTn [22]. It has been proposed that samples with macrotroponin, when analyzed using the Abbott and Ortho hs-cTnI assays, will have discordant results, defined as samples with a hs-cTnI concentration > 10 ng/L from at least one hs-cTnI assay (Ortho hs-cTnI/Abbott hs-cTnI) with fold differences < 0.33 or > 2.0 [23, 24]. The subset of residual plasma samples with hs-cTnI recoveries above the 40% threshold (no macrotroponin) all showed concordant results (Supplemental Table 1). Residual specimens were collected at Toronto General Hospital, Toronto and selected to reflect, as much as possible, the concentration of hs-cTnI in ERBB2 + breast cancer patients included in the pilot study. This cohort of patients had a median hs-cTnI concentration of 38 ng/L (range: 6 – 66 ng/L). All specimens were stored at -80 °C until further processing and were subjected to one freeze thaw cycle.

### Data analysis

All graphing was performed using R (https://www.R-project.org, accessed on 11/07 2023) with the following package: ggplot2 and GraphPad Prism version 8.4.3 (GraphPad Software). All statistical analysis was performed using GraphPad Prism version 8.4.3 (GraphPad Software).

## Results

### Patients

Of the 136 patients recruited into the EMBRACE-MRI study, 12 ERBB2 + breast cancer patients were included in this pilot study. Median patient age was 55 years (range: 30 – 69 years). Baseline characteristics are summarized Table 1 and Supplemental Table 2.

**Table 1:** Baseline characteristics of the pilot study cohort

Characteristic	No. (%)
Age, median (range), y	55 (30 – 69)
Blood pressure, median (range), mmHg	
Systolic	121 (100 – 159)
Diastolic	78 (69–101)
Heart rate, median (range), bpm	71 (60 – 91)
Diabetes	0 (0)
Hypertension	3 (25)
Dyslipidemia	2 (17)
Smoking	1 (8)
Baseline medications	
ACE inhibitor	1 (8)
Angiotensin receptor blocker	1 (8)
β-Blocker	1 (8)
Statin	1 (8)
Any cardiac medication	4 (33)
Systemic cancer therapy	
ACT-H	3 (25)
FEC-DH	9 (75)
Breast cancer stage	
1	2 (17)
2	9 (75)
3	1 (8)
4	0 (0)
Breast cancer side	
Right	10 (83)
Left	2 (17)

*ACT-H* Adriamycin, cyclophosphamide, paclitaxel, trastuzumab, *FEC-DH* 5-fluorouracil, epirubicin, cyclophophamide, docetaxel, trastuzumab

### Changes in high-sensitivity troponin concentrations

**Fig. 2:**
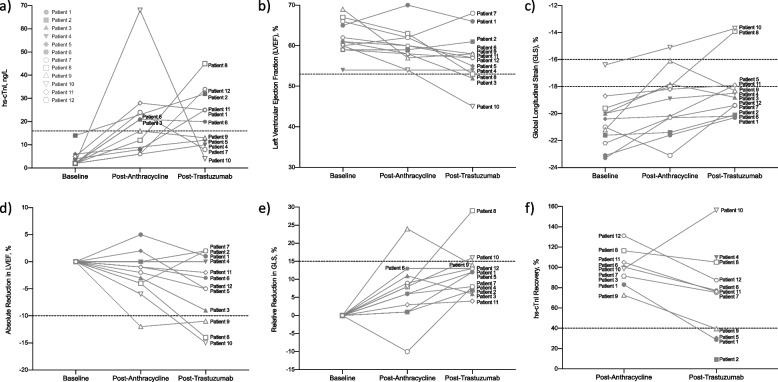
Troponin and imaging studies in patients with ERBB2 + breast cancer. **a** Hs-cTnI kinetics at baseline, post-anthracycline, and 3-months into trastuzumab therapy. Baseline hs-cTnI measurements were provided by the EMBRACE database and were performed on the ARCHITECT i2000 immunoassay analyzer (Abbott Diagnostics, Abbott Park, IL, USA). The dashed line indicates the hs-cTnI sex-specific 99th percentile of 16 ng/L. **b** LVEF kinetics at baseline, post-anthracycline, and 3-months into trastuzumab therapy. The dashed lines represent the cut-off for abnormal LVEF (LVEF < 53%). **c** GLS kinetics at baseline, post-anthracycline, and 3-months into trastuzumab therapy. The dashed lines indicate the normal (GLS < -18%), borderline (GLS -16% to -18%), and abnormal (GLS >—16%) ranges for GLS. **d** Absolute reduction in LVEF from baseline to ~ 2 months after therapy initiation (post-anthracycline), and 3-months into trastuzumab therapy. The dashed line indicates a 10% absolute reduction in LVEF from baseline. **d** Relative reduction in GLS from baseline to post-anthracycline and 3-months into trastuzumab therapy. The dashed line indicates a 15% relative reduction in GLS from baseline. **f** Recovery of hs-cTnI after protein G treatment in serum specimens collected post-anthracycline and 3-months into trastuzumab therapy. The dashed line indicates a hs-cTnI recovery cut-off of 40%, which was used to define the presence of macro-cTnI

The changes in hs-cTnI concentration for each patient at baseline, post-anthracycline, and 3-months into trastuzumab therapy are summarized in Fig. 2a and Supplemental Table 3. As shown in Fig. 2a, baseline hs-cTnI concentrations ranged from 2 to 14 ng/L (median: 2 ng/L and below the female-specific 99th percentile). At baseline, 8 patients (67%) had a hs-cTnI concentration at or below the assay’s limit of detection (LoD: 2 ng/L), while 4 patients (33%) had a hs-cTnI concentration between the assay’s LOD and the sex-specific 99th percentile. Nine patients were assessed post-anthracycline. As shown in Table 2, hs-cTnI concentrations ranged from 6 to 68 ng/L (median: 21 ng/L). In 5 patients (56%; Patients 3, 6, 7, 10, and 11), the hs-cTnI concentration increased from below the sex-specific 99th percentile at baseline to above the sex-specific 99th percentile. The median increase in hs-cTnI from baseline to post-anthracycline was 19 ng/L (range: 4 – 66 ng/L), reflecting a median relative percent increase of 156% (range: 67 – 189%). Eleven patients were assessed 3-months into trastuzumab therapy. As shown in Table 2, hs-cTnI concentrations ranged from 4 to 45 ng/L (median: 20 ng/L). In 6 patients (55%; Patients 1, 2, 6, 8, 11, and 12), the hs-cTnI concentration increased from below the sex-specific 99th percentile at baseline to above the sex-specific 99th percentile. The median rise in hs-cTnI levels from baseline to 3-months into trastuzumab therapy was 18 ng/L (range: 2 – 43 ng/L), which corresponded to a median relative percent increase of 133% (range: 67 – 183%). A total of eight patients were assessed at both timepoints, post-anthracycline and 3-months into trastuzumab therapy. Notably, 3 patients (~ 37%; Patients 1, 8, and 12) exhibited a significant rise in hs-cTnI levels post-anthracycline to 3-months into trastuzumab therapy (Fig. 2a).

**Table 2:** High-sensitivity cardiac troponin I and T (hs-cTnI and -cTnT) concentration and recovery post-anthracycline and 3-months into trastuzumab therapy

	Post-anthracycline (*n* = 9)	3-months into trastuzumab therapy (*n* = 11)
hs-cTnI (ng/L), median (range)	21 (6–68)	20 (4–45)
hs-cTnT (ng/L), median (range)	21 (7–34)	8^a^ (3–33)
Protein G hs-cTnI recovery (%), median (range)	99 (73–131)	76 (9–156)
Protein G hs-cTnT recovery (%), median (range)	112 (102–174)	110^a^ (34–228)

^a^Not assessed in all samples due to insufficient volume

### Changes in left ventricular ejection fraction (LVEF)

LVEF and GLS were used to assess cardiac function at baseline, and both follow-up time points. Of the 5 patients (Patients 3, 6, 7, 10, and 11) with hs-cTnI concentrations above the sex-specific 99th percentile post-anthracycline, all five had a normal LVEF (LVEF ≥ 53%) measured on the same day as the hs-cTnI, while Patient 3 (GLS: -17.8%) and Patient 10 (GLS: -15.1%) had borderline (borderline GLS: -16% to -18%) and abnormal (abnormal GLS: > -16%) GLS, respectively (Fig. 2b and c) [25]. None of these patients exhibited a significant reduction in LVEF or GLS post-anthracycline despite elevations in hs-cTnI (Fig. 2d and e), where a significant reduction in LVEF was defined as a > 10% absolute reduction in LVEF from baseline, while a significant reduction is GLS was defined as a > 15% relative reduction in GLS from baseline [3, 26]. Of the 6 patients (Patients 1, 2, 6, 8, 11, and 12) with hs-cTnI concentrations above the sex-specific 99th percentile 3-months into trastuzumab therapy, all six had a normal LVEF, while Patient 8 (GLS: -13.9%) and Patient 11 (GLS: -17.9%) had abnormal and borderline GLS, respectively (Fig. 2b and c). Moreover, Patient 8 exhibited a significant reduction in LVEF and GLS (Fig. 2d and e).

### Detection of macrotroponin and impact on cardiac function

IgG macro-cTnI was identified in 3 patients (Patients 1, 2, and 5) (Fig. 2f). The percentage recovery of hs-cTnI after protein G treatment post-anthracycline and 3-months into trastuzumab therapy are summarized in Table 2. In all 3 patients, macro-cTnI was detected in serum specimens collected 3-months into trastuzumab therapy. Two of the macro-cTnI positive patients (Patients 1 and 2) had a hs-cTnI value above the sex-specific 99th percentile (sex-specific 99th percentile: 16 ng/L) 3-months into trastuzumab therapy, while Patient 5 had a hs-cTnI value below the sex-specific 99th percentile. Macro-cTnI positivity was confirmed in two patients (Patients 2 and 5) by PEG (Patient 2: hs-cTnI recovery of 8%, Patient 5: 17% recovery). We were unable to confirm the presence of macro-cTnI in Patient 1 due to insufficient serum volume. Additionally, one patient (Patient 9) exhibited an hs-cTnI recovery at the threshold for defining macro-cTnI, with a hs-cTnI recovery of 40% after protein G treatment 3-months into trastuzumab therapy. PEG was performed to clarify the results in this borderline case, revealing a 4% hs-cTnI recovery, which confirmed the presence of macro-cTnI. Of note, Patients 1, 2, and 5 had normal LVEF (LVEF ≥ 53%) and GLS (GLS < -18%) 3-months into trastuzumab therapy. Also, none of these patients (Patients 1, 2, or 5) exhibited a significant reduction in LVEF or GLS despite having significant elevations in hs-cTnI. Patient 9 showed significant decline in cardiac function post-anthracycline, showing a 12% absolute reduction in LVEF from baseline and a 24% relative reduction in GLS. At 3-months into trastuzumab therapy, there was notable improvement, with an 11% absolute reduction in LVEF, and a 14% relative reduction in GLS from baseline. Overall, Patient 9 demonstrated an improvement in cardiac function from post-anthracycline to 3-months into trastuzumab therapy, despite persistent elevations in hs-cTnI (hs-cTnI baseline: < 2 ng/L, post-anthracycline: 16 ng/L, 3-months into trastuzumab therapy: 13 ng/L). Notably, both protein G (hs-cTnI recovery: 73%) and PEG (hs-cTnI recovery: 22%) treatments were negative for macro-cTnI in Patient 9 following anthracycline therapy, but 3-months into trastuzumab therapy, macro-cTnI was detected by PEG. In contrast, two macro-cTnI negative patients (Patients 8 and 10) with similar hs-cTnI elevations (hs-cTnI significantly elevated from baseline) had a significant absolute reduction in LVEF from baseline. Notably, Patient 10 showed a 15% absolute reduction in LVEF to a LVEF of 45% (baseline LVEF: 60%) 3-months into trastuzumab therapy. Moreover, Patients 8 and 10 also exhibited a significant relative reduction in GLS from baseline.

Overall, patients who were positive for macro-cTnI 3-months into trastuzumab therapy (Patients 1, 2, 5, and 9) tended to have similar LVEF and GLS compared to patients who were negative for macro-cTnI (*n* = 7) (Supplemental Fig. 2 a-b). Macro-cTnI positive patients had a mean LVEF of 60.0% (95%CI: 52.5 – 67.4%), while macro-cTnI negative patients had a mean LVEF of 56.0% (95%CI: 49.6 – 62.3%). The mean GLS in macro-cTnI positive and macro-cTnI negative patients was -19.2% (95%CI: -21.1 – -17.2%) and -17.6% (95%CI: -20.1 – -15.1%), respectively. Macro-cTnI positive patients also tended to have similar absolute reduction in LVEF from baseline and similar relative reduction in GLS from baseline, compared to macro-cTnI negative patients (Supplemental Fig. 2 c-d). The mean absolute reduction in LVEF from baseline in macro-cTnI positive and macro-cTnI negative patients was -3.3% (95%CI: -12.8 – 6.3%) and -5.3% (95%CI: -11.5 – 0.9%) respectively, whereas the mean relative reduction in GLS from baseline in macro-cTnI positive and macro-cTnI negative patients was 11.3% (95%CI: 6.5 – 16.0%) and 13.0% (95%CI: 5.5 – 20.5%), respectively.

Troponin T was measured in most of the available samples; post-anthracycline (*n* = 9) and 3-months into trastuzumab therapy (*n* = 10). Post-anthracycline, 7 patients (78%; Patients 3, 6, 7, 8, 9, 10, and 11) had a hs-cTnT concentration above the assay’s sex-specific 99th percentile (sex-specific 99th percentile: 9 ng/L), while 5 patients (out of 10; 50%; Patients 1, 5, 6, 8, and 12) had a hs-cTnT concentration above the assay’s sex-specific 99th percentile 3-months into trastuzumab therapy. Of note, 5 patients (Patients 3, 6, 7, 10, and 11) had both hs-cTnT and hs-cTnI concentrations above the sex-specific 99th percentile post-anthracycline, while 4 patients (Patients 1, 6, 8, and 12) had both hs-cTnT and hs-cTnI concentrations above the sex-specific 99th percentile 3-months into trastuzumab therapy. Of the 4 macro-cTnI positive patients, 2 patients (Patients 1 and 5) had a hs-cTnT concentration above the assay’s sex-specific 99th percentile (Patient 1: 10 ng/L, Patient 5: 19 ng/L). Patient 5 also exhibited low recovery of hs-cTnT (hs-cTnT recovery < 85%) after protein G treatment (Table 2), which may be suggestive of the presence of macro-cTnT.

## Discussion

Cardiac damage and dysfunction have been observed in some patients following anthracycline and trastuzumab treatment of ERBB2 + breast cancer patients. In patients receiving potentially cardiotoxic cancer therapy, cTn, in conjunction with cardiac imaging, is used for pre-treatment risk stratification, surveillance for cardiotoxicity during cancer treatment, and diagnosis of acute cardiac events [3, 5].

Interpretation of cTn results may be complicated by presence of macrotroponin. Macrotroponin (macro-cTn) is thought to consist of troponin in complex with anti-troponin immunoglobulin, particularly IgG, and can result in an increased cTn when present [11, 12, 27, 28]. It is thought that anti-troponin antibody forms a complex with troponin, increasing the half-life of troponin in circulation, resulting in higher-than-normal steady state troponin levels that may not indicate increased troponin release from the heart due to injury, although direct evidence for this hypothesis is sparse [13, 27, 29]. The presence of macro-cTn can also result in negative interference, possibly related to the masking of critical epitopes recognized by reagent antibodies used in hs-cTn assays [15], leading to falsely low troponin concentration. However, this is less frequently encountered in clinical practice, likely because it is more challenging to detect [28, 30–32].

Macro-cTnI was identified in 4 out of 12 patients. In all 4 patients, macro-cTnI was detected 3-months into trastuzumab therapy. Despite this, it is important to note that 2 (Patients 2 and 5) out of the 4 patients were not investigated for the presence of macrotroponin post-anthracycline due to insufficient sample volume. This is consistent with a recent study that identified macro-cTn in five breast cancer patients who had normal LVEF despite having hs-cTn elevations within the first 3-months of starting trastuzumab therapy following anthracycline chemotherapy [33].

One macro-cTnI positive patient also exhibited low recovery of hs-cTnT (< 85%) after protein G treatment, which may be suggestive of the presence of macro-cTnT [21]. There are few reports of concurrent macro-cTnI and macro-cTnT [12, 14, 21, 34–36]. Lam et al. previously described a subset of macro-cTnI patients with simultaneous low recovery in cTnT [21]. In addition, a recent case report described a patient with macrocomplexes for both creatinine kinase and cardiac troponin (macro-cTnI and -cTnT) [36].

Left ventricular ejection fraction (LVEF) and global longitudinal strain (GLS) are used for the detection of cancer therapy related cardiac dysfunction (CTRCD) during and after cardiotoxic treatment. Interestingly, none of the macro-cTnI positive patients (Patients 1, 2, 5, and 9) exhibited a significant reduction in LVEF or GLS despite having exhibited a significant increase in hs-cTnI from baseline. In contrast, two macro-cTnI negative patients (Patients 8 and 10) with similar hs-cTnI elevations (hs-cTnI significantly increased from baseline) had a significant absolute reduction in LVEF from baseline and a significant relative reduction in GLS in the same timeframe. This finding is consistent with previous reports that some patients with elevated cTn levels do not exhibit LV systolic dysfunction [8, 9].

The current study has several limitations. This was a pilot study and hence the number of patients included in this macrotroponin investigation is small. As such no conclusions should be made about the frequency of macrotroponin in anthracycline/trastuzumab-treated ERRB2 + breast cancer patients or the association between macrotroponin and patient outcome such as CTRCD. Second, the current study used protein G treatment to define the presence of macro-cTnI. We did not perform confirmatory methods, such as size exclusion gel filtration chromatography or sucrose density gradient ultracentrifugation [10, 13], due to serum volume restrictions. However, we did perform confirmatory studies with PEG. We did not measure hs-cTnI across multiple platforms in our cohort of anthracycline/trastuzumab-treated ERRB2 + breast cancer patients. Previous studies have noted discrepancies between commercially available hs-cTnI assays [21, 34, 35], largely due to the lack of standardization across platforms [37]. Macrotroponin may also contribute to these discrepancies due to differences in assay sensitivity to its presence [35, 38]. While we did not measure hs-cTnI across multiple platforms in this study, we did measure hs-cTnI using the VITROS High Sensitivity Troponin I assay on a subset of samples used to verify the 40% threshold for the detection of macro-cTnI. One macro-cTnI positive sample (hs-cTnI recovery < 40%) with discordant hs-cTnI results (Abbott vs. Ortho hs-cTnI assay) was identified. This aligns with findings by Warner and Marshall, who reported a higher rate of macrocomplexes with the Abbott hs-cTnI assay (a 2-site immunoassay) compared with the Ortho VITROS Troponin I ES assay (a 3-site immunoassay) [11]. These findings were further supported by a study demonstrating that the Ortho hs-cTnI assay showed significantly lower immunoreactivity to macrocomplexes compared to both the Siemens Dimension EXL hs-cTnI assay (another 3-site immunoassay) and the Abbott hs-cTnI assay [22]. In addition, detection of macrotroponin suggests presence of anti-troponin antibodies. However, auto-antibodies to troponin were not directly measured. As a result, it is not known if such antibodies were present at baseline or have developed over the course of therapy. More in depth studies are needed to understand the mechanism of antibody formation.

In conclusion, clinicians should be aware of macro-cTn interferences, as it can complicate interpretation of laboratory results. Future studies should aim to evaluate the prevalence of macro-cTn in a larger cohort of ERBB2 + breast cancer patients receiving cardiotoxic cancer therapies, while also assessing long-term health outcomes. Moreover, confirmatory methods for macro-cTn detection, including gel filtration chromatography or sucrose gradient ultracentrifugation, should be performed in cases where protein G and PEG is not informative.

## Supplementary Information

Supplementary Material 1. Supplemental Figure 1. Recovery of hs-cTnI and hs-cTnT after protein G in residual plasma samples. a) Histogram showing the distribution of hs-cTnI recovery after protein G treatment in 25 residual plasma samples. The dashed line indicates a hs-cTnI recovery cut-off of 40%, which was used to define the presence of macro-cTnI. b) Histogram showing the distribution of hs-cTnT recovery after protein G treatment in 25 residual plasma samples. The dashed line indicates a hs-cTnI recovery cut-off of 85%, which was used to define low hs-cTnT and possible macro-cTnT.

Supplementary Material 2. Supplemental Figure 2. LVEF and GLS measurement in ERBB2+ breast cancer patients with (*n* = 4) and without (*n* = 7) macro-cTnI 3-months into trastuzumab therapy. a) Mean LVEF (with 95% CIs) in patients 3-months into trastuzumab therapy with and without macro-cTnI. The dashed lines represent the cut-off for abnormal LVEF (LVEF < 53%). b) Mean GLS (with 95% CIs) in patients 3-months into trastuzumab therapy with and without macro-cTnI. The dashed lines indicate the normal (GLS < -18%), borderline (GLS -16% to -18%), and abnormal (GLS > - 16%) ranges for GLS. c) Mean absolute reduction in LVEF from baseline (with 95% CIs) in patients 3-months into trastuzumab therapy with and without macro-cTnI. The dashed line indicates a 10% absolute reduction in LVEF from baseline. d) Mean relative reduction in GLS from baseline (with 95% CIs) in patients 3-months into trastuzumab therapy with and without macro-cTnI. The dashed line indicates a 15% relative reduction in GLS from baseline.

Supplementary Material 3.

## Data Availability

No datasets were generated or analysed during the current study.

## Abbreviations

ERBB2: Epidermal growth factor 2
hs-cTn: High sensitivity cardiac troponin
