# Supplementary figures and images for: Macrotroponin interference and association with cardiotoxicity in patients receiving cardiotoxic breast cancer therapy: a pilot study
Source: Cardiooncology. 2025 Feb 14;11:18. doi: 10.1186/s40959-025-00314-9 (PMC11827144; doi:10.1186/s40959-025-00314-9)

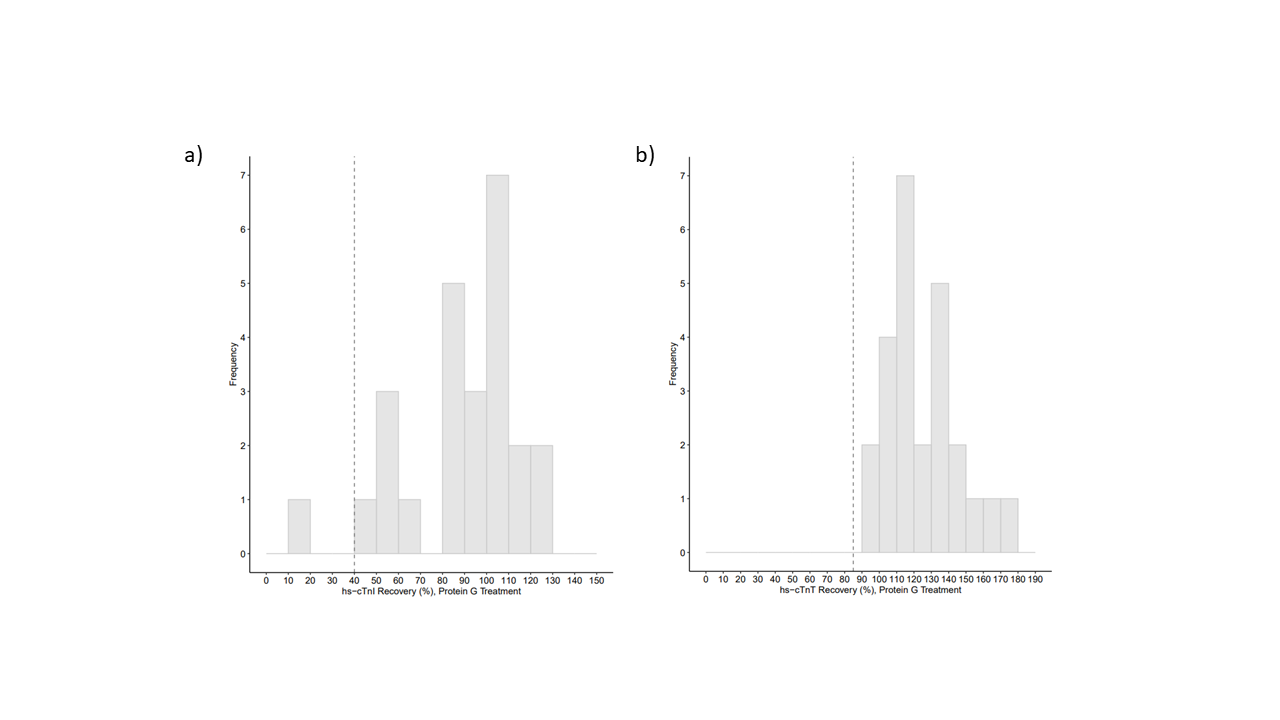

Supplement: Supplementary file 1 — Supplementary Material 1. Supplemental Figure 1. Recovery of hs-cTnI and hs-cTnT after protein G in residual plasma samples. a) Histogram showing the distribution of hs-cTnI recovery after protein G treatment in 25 residual plasma samples. The dashed line indicates a hs-cTnI recovery cut-off of 40%, which was used to define the presence of macro-cTnI. b) Histogram showing the distribution of hs-cTnT recovery after protein G treatment in 25 residual plasma samples. The dashed line indicates a hs-cTnI recovery cut-off of 85%, which was used to define low hs-cTnT and possible macro-cTnT. [file 40959_2025_314_MOESM1_ESM.tif]

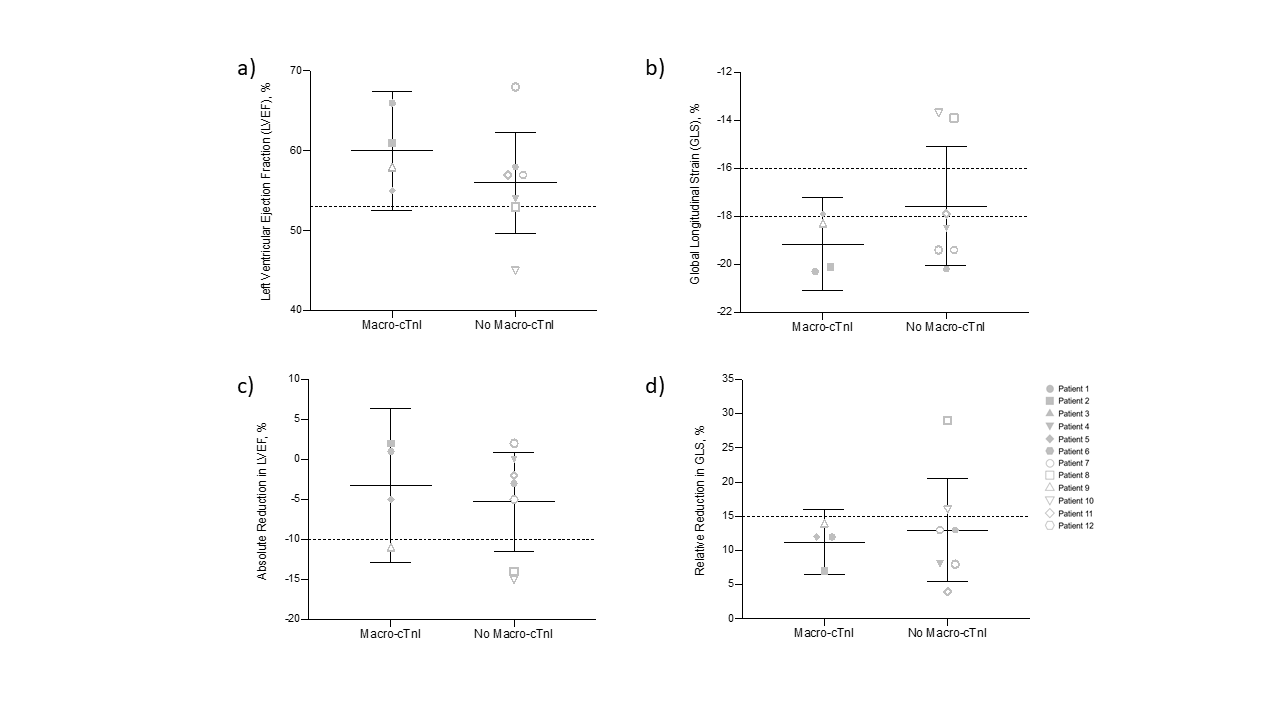

Supplement: Supplementary file 2 — Supplementary Material 2. Supplemental Figure 2. LVEF and GLS measurement in ERBB2+ breast cancer patients with (n = 4) and without (n = 7) macro-cTnI 3-months into trastuzumab therapy. a) Mean LVEF (with 95% CIs) in patients 3-months into trastuzumab therapy with and without macro-cTnI. The dashed lines represent the cut-off for abnormal LVEF (LVEF < 53%). b) Mean GLS (with 95% CIs) in patients 3-months into trastuzumab therapy with and without macro-cTnI. The dashed lines indicate the normal (GLS < -18%), borderline (GLS -16% to -18%), and abnormal (GLS > - 16%) ranges for GLS. c) Mean absolute reduction in LVEF from baseline (with 95% CIs) in patients 3-months into trastuzumab therapy with and without macro-cTnI. The dashed line indicates a 10% absolute reduction in LVEF from baseline. d) Mean relative reduction in GLS from baseline (with 95% CIs) in patients 3-months into trastuzumab therapy with and without macro-cTnI. The dashed line indicates a 15% relative reduction in GLS from baseline. [file 40959_2025_314_MOESM2_ESM.tif]
