# Supplementary material for: Macrotroponin interference and association with cardiotoxicity in patients receiving cardiotoxic breast cancer therapy: a pilot study
Source: Cardiooncology. 2025 Feb 14;11:18. doi: 10.1186/s40959-025-00314-9 (PMC11827144; doi:10.1186/s40959-025-00314-9)
Supplement: Supplementary file 3 — Supplementary Material 3. [file 40959_2025_314_MOESM3_ESM.docx]

**Supplemental Table 1.** Abbott and Ortho high-sensitivity cardiac troponin I concentrations and recoveries in de-identified residual plasma samples (n =25).

| **De-identified Samples** | **Sex** | **Abbott hs-cTnI, ng/L** | **Ortho hs-cTnI, ng/L** | **Fold Difference (Ortho hs-cTnI/Abbott hs-cTnI)** | **Abbott Protein G hs-cTnI Recovery (%)** | **Ortho Protein G hs-cTnI Recovery (%)** |
| --- | --- | --- | --- | --- | --- | --- |
| 1 | M | 57 | 38 | 0.67 | 90 | N/A |
| 2 | F | 28 | 13 | 0.46 | 89 | N/A |
| 3 | M | 30 | N/A | N/A | 102 | N/A |
| 4 | F | 43 | N/A | N/A | 62 | N/A |
| 5 | M | 42 | N/A | N/A | 54 | N/A |
| 6 | M | 10 | N/A | N/A | 123 | N/A |
| 7 | F | 43 | N/A | N/A | 106 | N/A |
| 8 | M | 50 | 26 | 0.52 | 115 | N/A |
| 9 | M | 51 | N/A | N/A | 105 | N/A |
| 10 | F | 58 | N/A | N/A | 107 | N/A |
| 11 | F | 66 | 25 | 0.38 | 108 | N/A |
| 12 | F | 52 | 26 | 0.50 | 95 | N/A |
| 13 | M | 19 | N/A | N/A | 130 | N/A |
| 14 | F | 41 | N/A | N/A | 82 | N/A |
| 15 | F | 6 | 1 | 0.17 | 56 | 178 |
| 16 | F | 7 | 1 | 0.14 | 42 | 146 |
| 17 | F | 13 | N/A | N/A | 101 | N/A |
| 18 | F | 37 | 20 | 0.54 | 92 | 80 |
| 19 | F | 38 | 42 | 1.11 | 51 | 169 |
| 20 | M | 24 | 21 | 0.88 | 114 | 119 |
| 21 | M | 19 | 28 | 1.47 | 88 | N/A |
| 22 | M | 45 | 75 | 1.67 | 109 | 95 |
| 23 | M | 50 | 52 | 1.04 | 91 | 82 |
| 24 | M | 32 | 27 | 0.84 | 88 | 94 |
| **25** | **F** | **19** | **1** | **0.05** | **17** | **163** |

Abbott female-specific 99^th^ percentile = 16 ng/L, Abbott male-specific 99^th^ percentile = 26 ng/L.

Ortho female-specific 99^th^ percentile = 9 ng/L, Ortho male-specific 99^th^ percentile = 13 ng/L.

**Bolded:** macro-cTnI positive sample with discordant hs-cTnI results (Abbott vs. Ortho hs-cTnI assay)

**Supplemental Table 2.** Baseline characteristics of the pilot study cohort.

| **Patient #** | **Age, years** | **SBP, mmHg** | **DBP, mmHg** | **HR, bpm** | **Breast cancer side** | **TNM stage** | **Cancer stage** | **Comorbidities** | **Baseline cardiac medications** | **Systemic**  **cancer therapy** | **Surgery** | **Radiation (Y/N)** |
| --- | --- | --- | --- | --- | --- | --- | --- | --- | --- | --- | --- | --- |
| 1 | 62 | 119 | 77 | 67 | Right | T2N0M0 | Stage 2a | HTN | Irbesartan 150mg OD | FEC-DH | Lumpectomy (right-sided) | Y |
| 2 | 58 | 109 | 69 | 83 | Right | T1cN0M0 | Stage 1a | HTN | Amlodipine 5mg OD Ramipril 10mg OD | FEC-DH | Lumpectomy (right-sided) | Y |
| 3 | 43 | 105 | 70 | 65 | Right | T2N1M0 | Stage 2b | GERD |  | FEC-DH | Lumpectomy (right-sided) | Y |
| 4 | 43 | 100 | 72 | 75 | Right | T1N0M0 | Stage 1a | None |  | FEC-DH | Mastectomy (right-sided) | Y |
| 5 | 54 | 135 | 86 | 60 | Right | T2N0M0 | Stage 2a | HTN |  | FEC-DH | Mastectomy (bilateral) | N |
| 6 | 51 | 106 | 70 | 60 | Right | T2N0M0 | Stage 2a | None |  | ACT-H | Mastectomy (right-sided) | N |
| 7 | 55 | 142 | 90 | 80 | Right | T2N0M0 | Stage 2a | None |  | FEC-DH | Lumpectomy (right-sided) | Y |
| 8 | 63 | 131 | 80 | 90 | Right | T2N0M0 | Stage 2a | DM  DLP | Metformin 250 mg BID  Crestor 10mg OD | ACT-H | Lumpectomy (right-sided) | Y |
| 9 | 42 | 112 | 73 | 66 | Left | T1cN1M0 | Stage 2a | None |  | FEC-DH | Mastectomy (bilateral) | Y |
| 10 | 61 | 149 | 101 | 91 | Left | T2N1M0 | Stage 2b | Palpitations Smoking | Bisoprolol 5mg OD | FEC-DH | Lumpectomy (right-sided) | Y |
| 11 | 69 | 159 | 82 | 66 | Right | T3N1M0 | Stage 3a | None |  | FEC-DH | Lumpectomy (right-sided) | Y |
| 12 | 30 | 123 | 78 | 86 | Right | T2N0M0 | Stage 2a | None |  | ACT-H | Mastectomy (right-sided) | N |

ACT-H, Adriamycin, cyclophosphamide, paclitaxel, trastuzumab; DBP, diastolic blood pressure; DLP, dyslipidemia; FEC-DH, 5-fluorouracil, epirubicin, cyclophophamide, docetaxel, trastuzumab; HR, heart rate; HTN, hypertension; OD, oral dose; SBP, systolic blood pressure

**Supplemental Table 3.** High-sensitivity cardiac troponin I and T (hs-cTnI and -cTnT) concentrations at baseline (prior to anthracycline therapy), post-anthracycline (~2 months after therapy initiation), and/or 3-months into trastuzumab therapy (~5 months after therapy initiation).

|  | **hs-troponin I (ng/L)** | | | **hs-troponin T (ng/L)** | | |
| --- | --- | --- | --- | --- | --- | --- |
|  | **Baseline*** | **Post-anthracycline** | **3-months into trastuzumab therapy** | **Baseline** | **Post-anthracycline** | **3-months into trastuzumab therapy** |
| Patient 1 | 4 | 8 | 25 | N/A | 8 | 10 |
| Patient 2 | 14 | N/A | 32 | N/A | N/A | 6 |
| Patient 3 | 2 | 21 | N/A | N/A | 21 | N/A |
| Patient 4 | 2 | N/A | 10 | N/A | N/A | 7 |
| Patient 5 | 6 | N/A | 12 | N/A | N/A | 19 |
| Patient 6 | 2 | 21 | 20 | N/A | 25 | 25 |
| Patient 7 | 2 | 24 | 8 | N/A | 23 | 5 |
| Patient 8 | 2 | 12 | 45 | N/A | 15 | 33 |
| Patient 9 | 2 | 16 | 13 | N/A | 10 | 3 |
| Patient 10 | 2 | 68 | 4 | N/A | 34 | 7 |
| Patient 11 | 5 | 28 | 25 | N/A | 29 | N/A |
| Patient 12 | 2 | 6 | 34 | N/A | 7 | 26 |

*****Baseline hs-cTnI measurements were provided by the EMBRACE database and were performed on the ARCHITECT i2000 immunoassay analyzer (Abbott Diagnostics, Abbott Park, IL, USA).

N/A: Not available
